# *Trans*-Selective Rhodium Catalysed Conjugate Addition of Organoboron Reagents to Dihydropyranones

**DOI:** 10.3390/molecules20046153

**Published:** 2015-04-08

**Authors:** Hannah J. Edwards, Sean Goggins, Christopher G. Frost

**Affiliations:** Department of Chemistry, University of Bath, Claverton Down, Bath BA2 7AY, UK; E-Mails: han.j.edwards@gmail.com (H.J.E.); sg297@bath.ac.uk (S.G.)

**Keywords:** boronic acids, conjugate addition, rhodium, tetrahydropyran

## Abstract

The selective synthesis of 2,6-*trans*-tetrahydropyran derivatives employing the rhodium catalysed addition of organoboron reagents to dihydropyranone templates, derived from a zinc-catalysed hetero-Diels-Alder reaction, is reported. The addition of both arylboronic acids and potassium alkenyltrifluoroborates have been accomplished in high yields using commercially-available [Rh(cod)(OH)]_2_ catalyst. The selective formation of the 2,6-*trans*-tetrahydropyran stereoisomer is consistent with a mechanism involving alkene association and carbometalation on the less hindered face of the dihydropyranone.

## 1. Introduction

**Figure 1:**
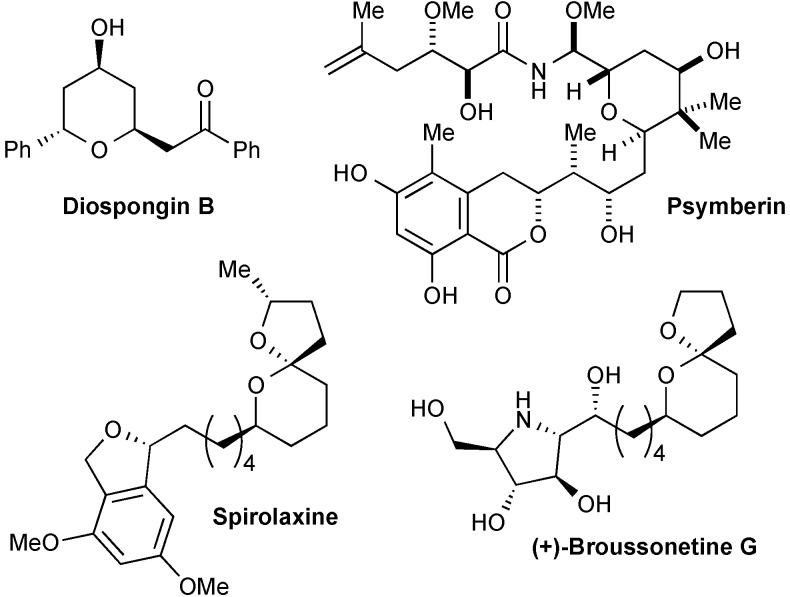
Representative examples of 2,6-*trans*-tetrahydropyran natural products.

The rhodium-catalysed conjugate addition of organometallic donors has evolved into a versatile tool for the assembly of complex molecules and intermediates in natural product synthesis [1,2,3,4,5,6]. The mechanistic and stereochemical aspects of the reaction have been thoroughly investigated for additions to prochiral substrates [7] and processes involving enantioselective protonation [8,9,10]. When the addition occurs to a chiral acceptor, the diastereoselectivity can be controlled by substrate [11], ligand [12] or organometallic donor [13]. Tetrahydropyran (THP) rings are a prevalent feature in natural products and such compounds frequently have important biological activities (Figure 1). In this context, the selective assembly of the 2,6-*trans*-tetrahydropyran subunit is a significant challenge [14]. A powerful methodology for the construction of 6-membered heterocycles is the hetero-Diels-Alder (HDA) cycloaddition [15]. This has been a key reaction for the synthesis of many THP containing natural products [16,17]. In this paper, we describe a general selective synthesis of 2,6-*trans*-tetrahydropyran derivatives employing the rhodium-catalysed addition of organoboron reagents to dihydropyranone templates derived from a HDA reaction.

## 2. Results and Discussion

**Scheme 1:**
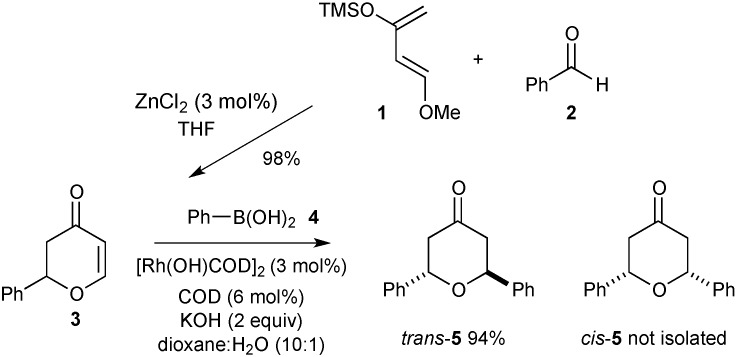
Catalytic synthesis of 2,6-*trans*-tetrahydropyran derivatives.

The required 5,6-dihydro-2*H*-pyranones, can be accessed using a zinc-catalysed HDA reaction of Danishefsky’s Diene **1** and an aldehyde heterodienophile [18]. As illustrated in Scheme 1, the use of benzaldehyde **2** results in an efficient synthesis of *rac*-2-phenyl-2,3-dihydro-pyran-4-one **3** in 98% isolated yield. Initial investigations into the rhodium-catalysed addition of phenylboronic acid **4** to **3** were carried out using 3 mol % [Rh(OH)(cod)]_2_ with additional ligand in dioxane:water (10:1) at 80 °C. ^1^H-NMR and chiral HPLC analysis of the isolated product indicated the formation of *rac*-2,6-*trans*-diphenyltetrahydropyran **5** in excellent yield. 

**Figure 2:**
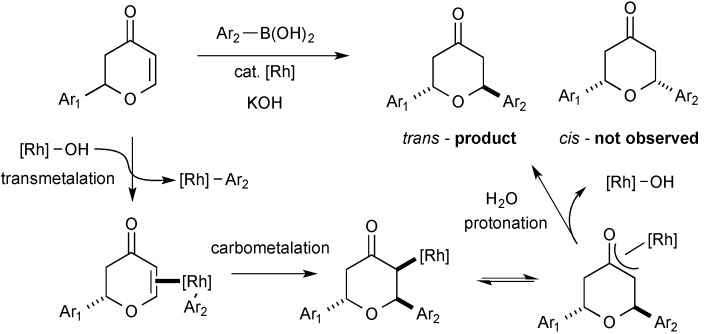
Mechanistic steps and origin of *trans*-selectivity.

A successful catalytic conjugate addition is dependent on an efficient transmetalation of the organoboronic acid to rhodium followed by carbometallation to afford an η^3^-oxa-π-allylrhodium complex that is protonated to afford the product. A number of detailed mechanistic studies for rhodium-catalysed conjugate addition to cyclic and acyclic, activated alkenyl species have been reported [1,2,3,4,5,6]. The selective formation of the 2,6-*trans*-tetrahydropyran stereoisomer is consistent with a mechanism involving alkene association and carbometalation on the less hindered face of the dihydropyranone, which affords the 2,6-*trans*-tetrahydropyran derivative on protonation of the rhodium oxa-π-allyl species (Figure 2).

**Figure 3:**
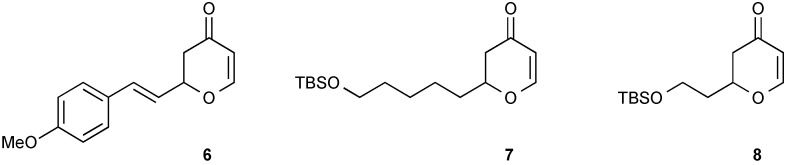
2,3-Dihydropyran-4-one substrates.

Following these successful initial results, a small range of functionalised 2,3-dihydropyran-4-one substrates were prepared using the zinc-catalysed HDA reaction (Figure 3). It is interesting to note that in many of the 2,6-*trans*-tetrahydropyran natural products, alkyl chains appear more frequently than aryl groups. Since the use of alkenylboronates in rhodium catalysed additions is well established, this tactical approach presents a synthetic opportunity to install a broad array of functionality from either HDA or conjugate addition. To establish useful scope for synthetic applications it was important to establish whether similar stereocontrol would be maintained in the addition of both aryl- and alkenylboronates. 

**Scheme 2:**
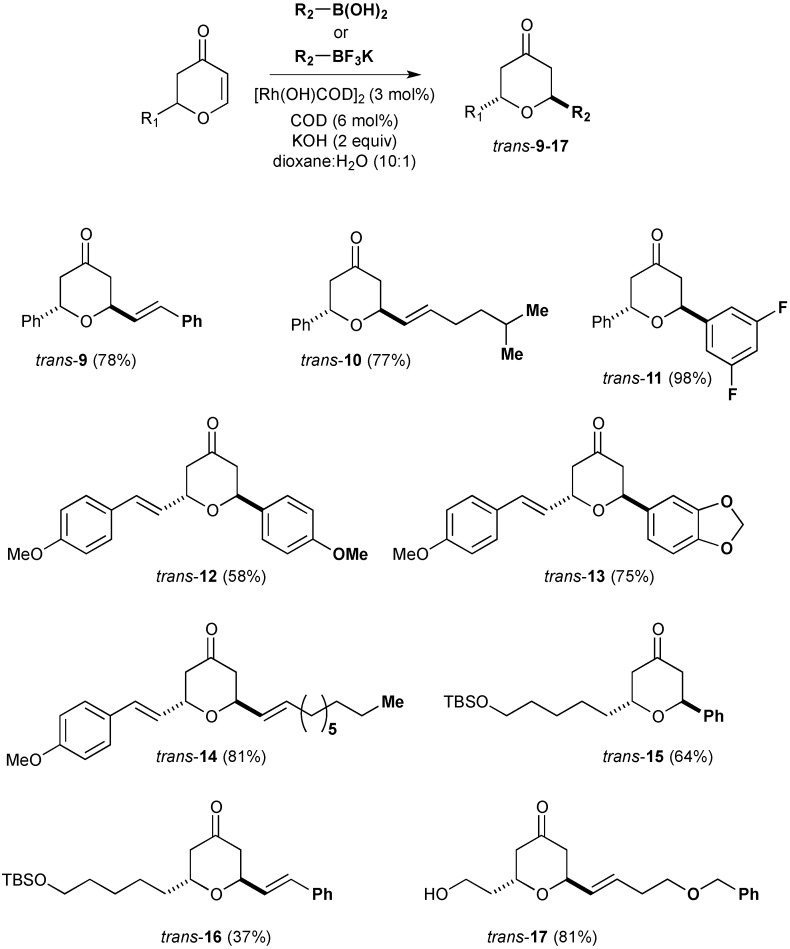
Catalytic synthesis of racemic 2,6-*trans*-tetrahydropyran derivatives.

We have previously noted that alkenyltrifluoroborate salts offer practical advantages in terms of stability and product yield in rhodium-catalysed conjugate addition reactions [19]. This is proposed to be due to the slow release of alkenylboronic acid and a concommitant reduction in competing protodeboronation pathways [20]. Therefore, rhodium-catalysed conjugate additions of arylboronic acids and potassium alkenyltrifluoroborates to the 2,3-dihydropyran-4-one substrates were explored. The optimised conditions for the addition of both arylboronic acids and potassium alkenyltrifluoroborates employed commercially-available [Rh(cod)(OH)]_2_ catalyst with added cycloctadiene ligand to limit catalyst decomposition. A diverse range of organoboronates were shown to successfully participate in the conjugate addition to 2,3-dihydropyran-4-ones affording the products **9**–**17** as the *trans* isomer (Scheme 2). 

**Scheme 3:**
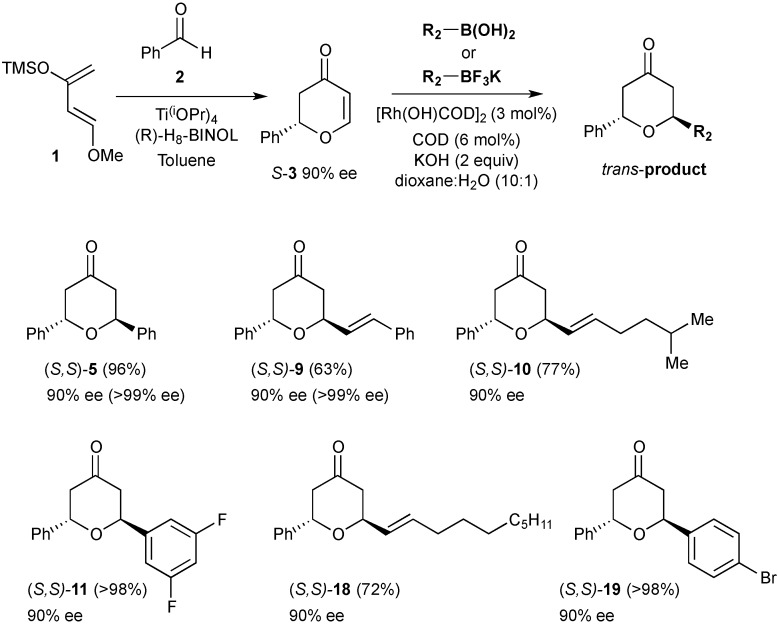
Catalytic, stereoselective additions to enantioenriched *S*-**3**.

The synthetic potential of this selective process was next explored with an enantiopure acceptor (Scheme 3). In this context, a suitable asymmetric synthesis of 2-phenyl-2,3-dihydro-pyran-4-one **3** was required. For catalytic asymmetric HDA reactions, a wide-range of chiral Lewis acid complexes have been successfully employed [21]. In particular, the use of Ti(OPr*^i^*)_4_ in combination with H_8_-BINOL offers excellent enantioselectivites and high yields for a wide range of dihydropyrones [22]. Under the reported conditions (*S*)-**3** was obtained in 85% yield and 90% ee. This would serve as a useful probe for the stereoselectivity of the catalytic conjugate addition and afford enantioenriched products. Pleasingly, the optimised conditions were effective for the addition of both arylboronic acids (products **5**, **11** and **19**) and potassium alkenyltrifluoroborates (products **9**, **10** and **18**). No erosion of enantiopurity was observed in the products indicating a highly stereoselective *trans* addition. The tetrahydropyran derivatives **5** and **9** were scaled-up and it was possible to recrystallise the products to amplify the ee to >99%. Confirmation of enantiopurity was established via NMR spectroscopic analysis, by the appearance of only one set of diastereotopic coupling signals in all environments and via chiral HPLC analysis.

## 3. Experimental Section

### 3.1. General Remarks

All reactions were carried out under an atmosphere of nitrogen, in oven-dried glassware unless otherwise stated. Dichloromethane, tetrahydrofuran (THF) and toluene were dried and degassed by passing through anhydrous alumina columns using an Innovative Technology Inc. PS-400-7 solvent purification system and stored under an atmosphere of argon prior to use. Proton, carbon, fluorine and phosphorus nuclear magnetic resonance (NMR) spectra were recorded on a Bruker Avance 300 or 400 spectrometer (^1^H-NMR at 300 or 400 MHz, ^13^C-NMR at 75.5 or 101 MHz, ^19^F-NMR at 376.5 MHz and ^31^P-NMR at 121.5 MHz). Chemical shifts for protons are reported downfield from tetramethylsilane and are referenced to residual protium in the solvent (^1^H-NMR: CHCl_3_ at 7.26 ppm, DMSO at 2.50 ppm, H_2_O at 4.79 ppm). Chemical shifts for carbons are reported in parts per million downfield from tetramethylsilane and are referenced to the carbon resonances of the solvent peak (^13^C-NMR: CDCl_3_ at 77.0 ppm, DMSO-*d*_6_ at 39.5 ppm). IR spectra were recorded on a Perkin-Elmer 1600 FT IR spectrophotometer, with absorbencies quoted as ν in cm^−1^. High resolution mass spectrometry (HRMS) was performed on a µTOF electrospray time-of-flight (ESI-TOF) mass spectrometer (Bruker Daltonik). Enantiomeric excesses were determined using HPLC performed on a perkin Elmer IBN series system using chiralcel columns with a UV detector at 254 nm. Melting points were obtained on a Bibby-Sterilin SMP10 melting point machine and are uncorrected. 

### 3.2. General Procedure for the Synthesis of Racemic Dihydropyranones

To a flame dried flask under an atmosphere of argon was added ZnCl_2_ (39 mg, 0.28 mmol, 3 mol %) and anhydrous diethyl ether (0.4 mL, 3 mol %). Anhydrous THF (100 mL) was added followed by freshly-purified aldehyde (9.42 mmol, 1.0 eq). The reaction was stirred for 10 min before dropwise addition of Danishefsky’s Diene (**1**) (2.7 mL, 14.13 mmol, 1.5 eq). The reaction was stirred overnight at room temperature and then filtered through celite and concentrated. The crude product was purified by flash column chromatography to afford the respective dihydropyranones.

### 3.3. Synthesis of Racemic 2-Phenyl-2,3-dihydropyran-4-one *(**3**)*

Freshly distilled benzaldehyde (0.96 mL, 9.42 mmol) was reacted under the standard procedure and the crude product purified by flash column chromatography (eluting with petrol:ethyl acetate 8:2) to afford the title compound as a red oil (1.2 g, 73% yield). 

R_f_ (petrol:ethyl acetate, 7:3); 0.29; ν_max_ (CH_2_Cl_2_)/cm^−1^; 3063 (C-H), 1722 (C=O), 1670 (C=C), 1593, 1583 (C=C), 1268, 1037 (C-O); δ_H_ (300 MHz; CDCl_3_); 7.48 (1H, dd, *J* = 6.0, 0.5 Hz, OC*H*), 7.44–7.35 (5H, m, Ar*H*), 5.52 (1H, dd, *J* = 6.0 Hz, 1.3 Hz, C*H*CO), 5.43 (1H, dd, *J* = 14.4, 3.5 Hz, CH_2_C*H*Ar), 2.91 (1H, dd, *J* = 16.9, 14.3 Hz, COC*H*H), 2.66 (ddd, *J* = 16.9, 3.5, 1.3 Hz, COH*H*); δ_C_ (75.5 MHz; CDCl_3_); 192.1, 163.2, 137.9, 129.0, 128.9, 126.2, 107.4, 81.1, 43.4; HRMS (ESI^+^) calcd for C_11_H_10_NaO_2_ [M+Na^+^] *m/z* 197.0579 found: *m/z* 197.0590.

All data in accordance with literature values [22].

### 3.4. Synthesis of Racemic 2-[2-(4-Methoxyphenyl)vinyl]-2,3-dihydropyran-4-one *(**6**)*

Recrystallised 4-methoxycinnamaldehyde (1.0 g, 6.17 mmol) was reacted under the standard procedure and the crude product purified by flash column chromatography (eluting with petrol:ethyl acetate 8:2) to afford the title compound as an orange solid (0.28g, 20% yield). 

R_f_ (petrol:ethyl acetate, 4:1); 0.29; δ_H_ (300 MHz; CDCl_3_); 7.40 (2H, d, *J* = 6.0 Hz, Ar*H*), 7.34 (1H, d, *J* = 8.5 Hz, OC*H*CH), 6.87 (2H, d, *J* = 8.5 Hz, Ar*H*), 6.70 (1H, d, *J* 15.9 Hz, ArC*H*CH), 6.16 (1H, dd, *J* = 15.9, 6.8 Hz, ArCHC*H*), 5.46 (1H, d, *J* = 6.0 Hz, COC*H*), 5.07–5.00 (1H, m, CH_2_C*H*O), 3.81 (3H, s, OC*H*_3_), 2.78–2.56 (2H, m, C*H*_2_CO); δ_C_ (75.5 MHz; CDCl_3_); 192.2, 163.2, 150.0, 133.6, 128.2, 128.1, 122.7, 114.1, 107.3, 80.1, 55.4, 42.1; HRMS (CI^+^) calcd for C_14_H_15_O_3_ [M+H]^+^ */z* 231.1016 found: *m/z*. 231.1059. 

All data in accordance with literature values [22].

### 3.5. Synthesis of Racemic 2-[5-(tert-Butyldimethylsilanyloxy)pentyl]-2,3-dihydropyran-4-one *(**7**)*

6-(*tert*-Butyldimethylsilanyloxy)hexanal (1.0 g, 4.09 mmol) was reacted under the standard procedure and the crude product purified by flash column chromatography (eluting with petrol:ethyl acetate 9:1) to afford the title compound as a yellow oil (0.990 g, 81% yield). 

R_f_ (petrol:ethyl acetate, 4:1); 0.56; δ_H_ (300 MHz; CDCl_3_); 7.35 (1H, d, *J* = 5.9 Hz, CHC*H*O), 5.39 (1H, dd, *J* = 5.9, 1.0 Hz, C*H*CHO), 4.39 (1H, ddt, *J* = 12.7, 7.7, 4.6 Hz, CH_2_C*H*CH_2_), 3.61 (2H, t, *J* = 6.3 Hz, C*H*_2_OSi), 2.52 (1H, dd, *J* = 16.7, 12.9 Hz, COC*H*H), 2.42 (1H, ddd, *J* = 16.7, 4.3, 1.0 Hz, COCH*H*), 1.89–1.64 (2H, m, CHCH_2_C*H*_2_), 1.58–1.49 (2H, m, C*H*_2_CH_2_OSi), 1.48–1.33 (4H, m, (C*H*_2_)_2_(CH_2_)_2_OSi), 0.89 (9H, s, SiC(C*H*_3_)_3_), 0.04 (6H, s, Si(C*H*_3_)_2_); δ_C_ (75.5 MHz; CDCl_3_); 192.9, 163.4, 107.0, 79.6, 63.0, 41.9, 34.5, 32.7, 26.0, 25.7, 24.7, 18.4, −5.2; HRMS (ESI^+^) calcd for C_16_H_30_O_3_Si [M+H]^+^ *m/z* 299.2037 found: *m/z* 299.2052.

All data in accordance with literature values [23]. 

### 3.6. Synthesis of Racemic 2-[2-(tert-Butyl-dimethyl-silanyloxy)-ethyl]-2,3-dihydropyran-4-one *(**8**)*

3-(*tert*-butyldimethylsilanyloxy)propionaldehyde (1.0 g, 9.336 mmol) was reacted under the standard procedure and the crude product purified by flash column chromatography (eluting with petrol:ethyl acetate 4:1) to afford the title compound as a yellow oil (0.400 g, 39% yield). 

R_f_ (petrol:ethyl acetate, 4:1); 0.51; δ_H_ (300 MHz; CDCl_3_); 7.35 (1H, d, *J =* 6.0 Hz, OC*H*), 5.41 (1H, dd, *J* = 6.0, 1.0 Hz, C*H*CO), 4.67–4.57 (1H, m, OC*H*CH_2_), 3.85–3.71 (2H, m, OC*H*_2_), 2.63–2.43 (2H, m, COC*H*_2_), 2.07–1.96 (1H, m, CHC*H*HCH_2_), 1.90–1.79 (1H, m, CHCH*H*CH_2_), 0.89 (9H, s, SiC(C*H*_3_)_3_), 0.05 (6H, s, Si(C*H*_3_)_2_); δ_C_ (75.5 MHz; CDCl_3_); 192.7, 163.2, 107.2, 58.4, 42.1, 37.4, 26.0, 18.4, −5.32, −5.36; HRMS (ESI^+^) calcd for C_13_H_25_O_3_ [M+H]^+^ *m/z* 257.1572 found: *m/z* 257.1536. 

All data in accordance with literature values [23]. 

### 3.7. Synthesis of (S)-2-Phenyl-2,3-dihydropyran-4-one ((S)-**3**)

A mixture of (*R*)-H_8_-BINOL (0.610 g, 2.07 mmol) and Ti(O^i^Pr)_4_ (0.56 mL, 1.884 mmol) with activated 4 Å molecular sieves (4.54 g) in anhydrous toluene (38 mL) under an inert atmosphere was heated at 35 °C for 1 h. The yellow mixture was cooled to room temperature and freshly distilled benzaldehyde (0.96 mL, 9.42 mmol, 1.0 eq) added. After stirring for 10 min the mixture was cooled to 0 °C and Danishefsky’s diene (11.3 mmol, 1.2 eq) was added. The reaction was stirred at 0 °C for 24 h and then treated with trifluoroacetic acid (0.1 mL). After stirring for a further 15 min at 0 °C, NaHCO_3_ (10 mL) was added and the reaction stirred for 10 min and then filtered through a plug of celite. The organic layer was then separated and the aqueous extracted with diethylether (3 × 25 mL). The combined organic extracts were dried over Na_2_SO_4_ and concentrated *in vacuo*. The crude product was purified by flash chromatography (eluting with petrol:ethyl acetate 8:2) to afford the title compound as a red oil (1.2 g, 73% yield). The chromatographed material was determined to be in 90% ee by chiral HPLC analysis (Chiralcel OD, 9:1 Hexanes: propan-2-ol, 1.0 mL·min^−1^, t_R_ = 11.22 min (major) and 13.23 min (minor). 

R_f_ (petrol:ethyl acetate, 7:3); 0.29; ${\left[\alpha \right]}_{D}^{20}$ = +81° (*c* = 0.8, CHCl_3_); ν_max_ (CH_2_Cl_2_)/cm^−1^; 3063 (C-H), 1722 (C=O), 1670 (C=C), 1593, 1583 (C=C), 1268, 1037 (C-O)δ_H_ (300 MHz; CDCl_3_); 7.48 (1H, dd, *J* = 6.0, 0.6 Hz, OC*H*CH), 7.44–7.38 (5H, m, Ar*H*), 5.53 (1H, dd, *J* = 6.0 Hz, 1.2 Hz, C*H*CO), 5.43 (1H, dd, *J* = 14.4, 3.5 Hz, CH_2_C*H*Ar), 2.91 (1H, dd, *J* = 17.0, 14.4 Hz, COC*H*H), 2.66 (ddd, *J* = 17.0, 3.5, 1.3 Hz, COH*H*); δ_C_ (75.5 MHz; CDCl_3_); 192.2, 163.2, 137.9, 129.0, 128.9, 126.2, 107.5, 81.2, 43.5; HRMS (ESI^+^) calcd for C_11_H_10_NaO_2_ [M+Na]^+^ *m/z* 197.0579 found: *m/z* 197.0590. 

All data in accordance with literature values [22]. 

### 3.8. General Procedure for the Rhodium-Catalysed Conjugate Additions to Dihydropyranones

An oven dried, 24 mL screw-capped vial equipped with a rubber septum was charged with organoboron reagent (0.228 mmol, 2.0 eq), [Rh(OH)(cod)]_2_ (0.0016 g, 0.00342 mmol, 3 mol %), cyclooctadiene (0.007 g, 0.00684 mmol) and potassium hydroxide (0.009 g, 0.228 mmol). The reaction vessel was purged with argon and dioxane (0.5 mL) and water (0.05 mL) were subsequently added by syringe. The red solution was stirred for 15 minutes at room temperature, before the addition of dihydropyranone (0.114 mmol, 1.0 eq). The reaction was transferred to a preheated hotplate at 80 °C for 20 h. Upon completion, the crude reaction mixture was taken up in diethyl ether (5 mL) and filtered through a short plug of silica (elution; diethyl ether) and the solvent removed *in vacuo*. The crude residue was purified by flash column chromatography on silica gel to afford the desired compounds. 

### 3.9. Synthesis (2S,6S)-Diphenyltetrahydropyran-4-one *(**5**)*

Phenylboronic acid (0.210 g, 1.72 mmol) was treated with (*S*)-2-phenyl-2,3-dihydropyran-4-one ((*S*)-**3**) (0.150 g, 0.86 mmol) under the standard conditions. The crude residue was purified by flash column chromatography on silica gel (eluting with petrol:ethyl acetate 9:1) to afford the title compound as a white solid (0.207 g, 96% yield).

R_f_ (petrol:ethyl acetate, 4:1); 0.47; ${\left[\alpha \right]}_{D}^{20}$ = −16° (*c* = 1, CHCl_3_); ν_max_ (CH_2_Cl_2_)/cm^−1^; 3067, 3066, 2974, 2886 (C-H), 1714 (C=O), 1601 (C=C aryl), 1133 (C-O); δ_H_ (300 MHz; CDCl_3_); 7.27–7.26 (5H, m, Ar*H*), 7.05 (2H, m, C*H*OC*H*), 6.87 (2H, dd, *J* = 14.6, 6.6 Hz, CH*H*COC*H*H), 6.81 (2H, dd, *J* = 15.0, 5.0 Hz, C*H*HCOCH*H*); δ_C_ (75.5 MHz; CDCl_3_); 206.8, 139.9, 128.8, 128.2, 126.8, 73.6, 46.4; HRMS (ESI^+^) calcd for C_17_H_16_NaO_2_ [M+Na]^+^ *m/z* 275.1048 found: *m/z*. 275.1029; HPLC (Chiralcel ODH, 97:3 Hexanes:propan-2-ol, 0.5 mL·min^−1^, t_R_ = 11.07 min (major) and 13.11 min (minor).

### 3.10. Synthesis of (2S,6S)-2-Phenyl-6-styryltetrahydropyran-4-one *(**9**)*

Potassium (*E*)-styryltrifluoroborate (0.907 g, 4.32 mmol) was reacted with (*S*)-2-phenyl-2,3-dihydropyran-4-one ((*S*)-**3**) (0.20 g, 1.148 mmol) under the standard conditions. The crude residue was purified by flash column chromatography on silica gel (eluting with petrol:ethyl acetate 9:1) to afford the title compound as a white solid (0.20 g, 63% yield).

R_f_ (petrol:ethyl acetate, 4:1); 0.5; ${\left[\alpha \right]}_{D}^{20}$ = −77° (*c* = 1, CHCl_3_), ν_max_ (neat)/cm^−1^; 3035, 2979, 2882 (C-H), 1720 (C=O), 1658 (C=C), 1600, 1579 (C=C aryl), 1231, 1048 (C-O); δ_H_ (300 MHz; CDCl_3_); 7.34–7.17 (10H, m, Ar*H*), 6.53 (1H, dd, *J* = 16.3, 1.4 Hz, ArC*H*), 6.23 (1H, dd, *J* = 16.3, 5.0, ArCHC*H*), 5.12 (1H, dd, *J* = 7.4, 5.0 Hz, ArC*H*O), 4.83 (1H, ddd, *J* = 10.7, 5.2, 1.4 Hz, CHC*H*O), 2.79–2.63 (4H, m, C*H*_2_COC*H*_2_); δ_C_ (75.5 MHz; CDCl_3_); 206.5, 140.3, 136.0, 133.5, 128.8, 128.7, 128.3, 128.2, 127.8, 126.7, 126.5, 73.6, 72.9, 47.8, 45.4; HRMS (ESI^+^) calcd for C_19_H_18_NaO_2_ [M+Na]^+^ *m/z* 301.1204 found: *m/z*. 301.1177; HPLC (Chiralcel ODH; 95.5 Hexanes:propan-2-ol, 1.0 mL·min^−1^, t_R_ = 13.37 min (major) and 21.93 min (minor).

### 3.11. Synthesis of (2S, 6S)-2-(5-Methylhex-1-enyl)-6-phenyltetrahydropyran-4-one *(**10**)*

Potassium (*E*)-trifluoro(5-methyl-hex-1-enyl)borate (0.047 g, 0.23 mmol) was reacted with (*S*)-2-phenyl-2,3-dihydropyran-4-one ((*S*)-**3**) (0.020 g, 0.115 mmol) under the standard conditions. The crude residue was purified by flash column chromatography on silica gel (eluting with petrol:ethyl acetate 9:1) to afford the title compound as a yellow oil (0.024 g, 77% yield).

R_f_ (petrol:ethyl acetate, 4:1); 0.78; ν_max_ (neat)/cm^−1^; 3068, 2955, 2870 (C-H), 1706 (C=O), 1648 (C=C), 1602 (C=C aryl), 1268, 1069 (C-O); δ_H_ (300 MHz; CDCl_3_); 7.38–7.27 (5H, m, Ar*H*), 5.69 (1H, dtd, *J* = 15.7, 6.3, 0.9 Hz, CH_2_C*H*CH), 5.57 (1H, ddt, *J* = 15.7, 5.0, 1.0 Hz, CH_2_CHC*H*), 5.11 (1H, dd, *J =* 7.4, 5.3 Hz, ArC*H*O), 4.70 (1H, dd, *J* = 9.7, 4.8 Hz, CHC*H*O), 2.76 (1H, dd, *J* = 14.4, 5.7, C*H*HCOCHH), 2.70 (2H, d, *J* = 5.4 Hz, CH*H*COC*H*H), 2.60 (1H, ddd, *J =* 14.4, 4.6, 1.0 Hz, CHHCOCH*H*), 2.10–2.03 (2H, m, CH_2_CH_2_CH), 1.53 (1H, nonet, *J* = 6.6 Hz, (CH_3_)_2_C*H*), 1.29–1.24 (2H, m, (CH_3_)_2_CHC*H*_2_), 0.88 (6H, d, *J* = 6.6 Hz, (C*H*_3_)_2_CH); δ_C_ (75.5 MHz; CDCl_3_); 206.9, 140.5, 136.1, 128.1, 126.4, 73.1, 72.9, 48.0, 45.4, 38.1, 30.4, 27.6, 22.5; HPLC (Chiralcel AD; 98:2 Hexanes:propan-2-ol, 1.0 mL·min^−1^, t_R_ = 6.85 min (major) and 15.91 min (minor).

### 3.12. Synthesis of (2S,6S)-2-(3,5-Difluorophenyl)-6-phenyltetrahydropyran-4-one *(**11**)*

3,5-difluorophenylboronic acid (0.045 g, 0.287 mmol) was reacted with (*S*)-2-phenyl-2,3-dihydropyran-4-one ((*S*)-**3**) (0.025 g, 0.144 mmol) under the standard conditions. The crude residue was purified by flash column chromatography on silica gel (eluting with petrol:ethyl acetate 9:1) to afford the title compound as a yellow solid (0.038 g, 98% yield).

R_f_ (petrol:ethyl acetate, 4:1); 0.6; δ_H_ (300 MHz; CDCl_3_); 7.42–7.30 (5H, m, Ar*H*), 6.90 (2H, ddd, *J* = 8.2, 2.2, 0.7 Hz, FCC*H*CC*H*CF), 6.75 (1H, tt, *J* = 8.8, 2.3 Hz, CFC*H*CF), 5.34 (1H, t, *J* = 5.7 Hz, OC*H*), 4.97 (1H, dd, *J =* 6.82, 5.64 Hz, OC*H*), 2.93 (2H, ddd, *J* = 14.5, 5.7, 0.9 Hz, C*H*HCOCH*H*), 2.85–2.72 (2H, m, CH*H*COC*H*H); δ_C_ (75.5 MHz; CDCl_3_); 205.7, 144.2, 139.2, 128.9, 128.5, 127.0, 109.7, 109.3, 103.5, 74.2, 72.3, 46.8, 45.8; HPLC (Chiralcel AD: 99:1 Hexanes:propan-2-ol, 0.5 mL·min^−1^, t_R_ = 37.30 min (major) and 48.68 min (minor).

### 3.13. Synthesis of trans-2-(4-Methoxyphenyl)-6-[2-(4-methoxyphenyl)-vinyl]-tetrahydropyran-4-one *(**12**)*

4-methoxyphenylboronic acid (0.026 g, 0.174 mmol) was reacted with 2-[2-(4-Methoxyphenyl)-vinyl]-2,3-dihydropyran-4-one (**6**) (0.020 g, 0.0869 mmol) under the standard conditions. The crude residue was purified by flash column chromatography on silica gel (eluting with petrol:ethyl acetate 8:2) to afford the title compound as a yellow oil (0.017 g, 58% yield).

R_f_ (petrol:ethyl acetate, 4:1); 0.26; δ_H_ (300 MHz; CDCl_3_); 7.32 (4H, dd, *J* 8.8, 2.2 Hz, ArH, 6.88 (4H, dd, *J* = 11.6, 8.8 Hz, ArH), 6.52 (1H, d, *J* = 16.2, 5.3 Hz, ArCH), 6.15 (1H, d, *J* = 16.2 Hz, 5.3 Hz, ArCHC*H*), 5.17 (1H, dd, *J* = 7.2, 4.9, ArC*H*O), 4.81 (1H, ddd, *J* = 9.4, 5.3, 1.4, CHC*H*O), 3.81 (6H, s, OC*H*_3_, OC*H*_3_), 2.87–2.63 (4H, m, CH_2_COCH_2_); HRMS (ESI^+^) calcd for C_21_H_22_NaO_4_ [M+Na]^+^ *m/z* 361.1416 found: *m/z*. 361.1404. 

### 3.14. Synthesis of trans-2-Benzo[1,3]dioxol-5-yl-6-[2-(4-methoxyphenyl)vinyl]-tetrahydropyran-4-one *(**13**)*

Benzo-[1,3]dioxol-5-ylboronic acid (0.029 g, 0.174 mmol) was reacted with 2-[2-(4-methoxyphenyl)-vinyl]-2,3-dihydropyran-4-one (6) (0.020 g, 0.0869 mmol) under the standard conditions. The crude residue was purified by flash column chromatography on silica gel (eluting with petrol:ethyl acetate 8:2) to afford the title compound as a yellow oil (0.032 g, 75% yield).

R_f_ (petrol:ethyl acetate, 4:1); 0.38; ν_max_ (neat)/cm^−1^; 2906, 2862 (C-H), 1715 (C=O), 1641 (C=C), 1606, 1577, 1511 (C=C aryl), 1246, 1033 (C-O); δ_H_ (300 MHz; CDCl_3_); 7.33 (3H, d, *J* 8.8 Hz, Ar*H*), 6.89 (2H, d, *J* = 11.7 Hz, Ar*H*), 6.82 (2H, d, *J* = 11.5 Hz, ArH), 6.53 (1H, d, *J* = 16.2 Hz, ArC*H*CH), 6.14 (1H, dd, *J* = 16.4, 5.5 Hz, ArCHC*H*), 5.96 (2H, s, OC*H*_2_O), 5.10 (1H, dd, *J* = 7.0, 5.4 Hz, ArC*H*O), 4.84 (1H, ddd, *J* = 10.6, 5.3, 1.2 Hz, CHC*H*O), 3.81 (3H, s, ArOC*H*_3_), 2.83–2.65 (4H, m, C*H*_2_COC*H*_2_); HRMS (ESI^+^) calcd for C_21_H_20_NaO_5_ [M+H]^+^ *m/z* 375.1208 found: *m/z*. 375.1192.

### 3.15. Synthesis of trans-2-Dec-1-enyl-6-[2-(4-methoxyphenyl)vinyl]-tetrahydropyran-4-one *(**14**)*

Potassium decenyl trifluoroborate (0.043 g, 0.174 mmol) was reated with 2-[2-(4-methoxyphenyl)-vinyl]-2,3-dihydropyran-4-one (**6**) (0.020 g, 0.0869 mmol) under the standard conditions. The crude residue was purified by flash column chromatography on silica gel (eluting with petrol:ethyl acetate 8:2) to afford the title compound as a yellow oil (0.026 g, 81% yield).

R_f_ (petrol:ethyl acetate, 4:1); 0.5; ν_max_ (CH_2_Cl_2_)/cm^−1^; 2925, 2855 (C-H), 1718 (C=O), 1610, 1514 (C=C aryl), 1250 (C-O); δ_H_ (300 MHz; CDCl_3_); 7.32 (2H, d, *J* = 8.7 Hz, Ar*H*), 6.85 (2H, d, *J* = 8.7 Hz, Ar*H*), 6.53 (1H, d, *J* = 16.1 Hz, ArC*H*CH), 6.12 (1H, dd, *J* = 16.1, 5.6 Hz, ArCHC*H*), 5.71 (1H, dt, *J* = 16.0, 6.6 Hz, CH_2_C*H*CH), 5.55 (1H, dd, *J* = 15.7, 5.5 Hz, CH_2_CHC*H*), 4.80 (1H, dd, *J* = 10.7, 5.1 Hz, C*H*O), 4.67 (1H, dd, *J* = 10.7, 5.6 Hz, C*H*O), 3.08 (3H, s, OC*H*_3_), 2.69–2.47 (4H, m, C*H*_2_COC*H*_2_), 2.05 (2H, q, *J* = 6.9 Hz, C*H*_2_CHCH), 1.37–1.26 (12H, m, CH_3_(C*H*_2_)_6_), 0.88 (3H, t, *J =* 6.6 Hz, CH_3_(C*H*_2_)_6_); δ_C_ (75.5 MHz; CDCl_3_); 206.7, 159.9, 135.4, 132.4, 128.6, 127.9, 126.0, 125.7, 114.1, 72.6, 72.5, 55.4, 46.3, 32.5, 32.0, 29.5, 29.3, 29.3, 29.0, 22.8, 14.2; HRMS (ESI^+^) calcd for C_24_H_35_O_3_ [M+H]^+^ *m/z* 371.2586 found: *m/z* 371.2588.

### 3.16. Synthesis of trans-2-[5-(tert-Butyl-dimethylsilanyloxy)-pentyl]-6-phenyltetrahydropyran-4-one *(**15**)*

Phenylboronic acid (0.021 g, 0.168 mmol) was reacted with (*S*)-2-[5-(*tert*-Butyl-dimethylsilanyloxy)-pentyl]-2,3-dihydropyran-4-one (**7**) (0.025 g, 0.0838 mmol) under the standard conditions. The crude residue was purified by flash column chromatography on silica gel (eluting with petrol:ethyl acetate 9:1) to afford the title compound as a colourless oil (0.020 g, 64% yield).

R_f_ (petrol:ethyl acetate, 4:1); 0.72; δ_H_ (300 MHz; CDCl_3_); 7.41–7.28 (5H, m, Ar*H*), 5.21 (1H, t, *J* = 5.7 Hz, ArC*H*O), 3.98–3.90 (1H, m, OC*H*CH_2_), 3.57 (2H, t, *J* = 1.56 Hz, C*H*_2_OSi), 2.88–2.74 (2H, m, COC*H*_2_), 2.57 (1H, ddd, *J* = 14.4, 4.5, 1.1 Hz, COC*H*H), 2.34 (1H, dd, *J* = 14.4, 7.3, 1.1 Hz, COCH*H*), 1.54–1.40 (4H, m, C*H*_2_C*H*_2_), 1.37–1.27 (4H, m, C*H*_2_C*H*_2_), 0.88 (9H, s, C(C*H*_3_)_3_), 0.03 (6H, s, Si(C*H*_3_)_2_); δ_C_ (75.5 MHz; CDCl_3_); 207.3, 140.2, 128.6, 128.0, 126.8, 63.1, 47.2, 46.2, 34.55, 32.72, 25.9, 25.6, 25.1, 18.4, −5.26.

### 3.17. Synthesis of trans-2-[5-(tert-Butyldimethylsilanyloxy)pentyl]-6-styryltetrahydropyran-4-one *(**16**)*

Potassium (*E*)-styryl trifluoroborate (0.028 g, 0.13 mmol) was reacted with (*S*)-2-[5-(*tert*-Butyl-dimethyl-silanyloxy)-pentyl]-2,3-dihydropyran-4-one (**7**) (0.020 g, 0.067 mmol) under the standard conditions. The crude residue was purified by flash column chromatography on silica gel (eluting with petrol:ethyl acetate 9:1) to afford the title compound as a colourless oil (0.010 g, 37% yield).

R_f_ (petrol:ethyl acetate, 4:1); 0.62; ν_max_ (neat)/cm^−1^; 2931, 2858 (C-H), 1713 (C=O), 1623 (C=C), 1579, 1569 (C=C aryl), 1251, 1049 (C-O); δ_H_ (300 MHz; CDCl_3_); 7.40–7.27 (5H, Ar*H*), 6.56 (1H, dd, *J* = 16.1, 1.2, ArC*H*), 6.23 (1H, dd, *J* = 11.2, 5.2, ArCHC*H*), 4.87 (1H, dd, *J* = 9.6, 4.5 Hz, CHC*H*O), 4.12–4.03 (1H, m, OC*H*CH_2_), 3.59 (2H, t, *J* = 6.4 Hz, C*H*_2_OTBS), 2.68 (2H, qd, *J* = 14.3, 5.4 Hz, COC*H*_2_), 2.49 (1H, ddd, *J* = 14.2, 4.0, 1.2 Hz, COC*H*H), 2.29 (1H, dd, *J* = 13.9, 8.2Hz, COCH*H*), 1.55–1.46 (4H, m, C*H*_2_C*H*_2_), 1.43–1.34 (4H, m, C*H*_2_C*H*_2_), 0.88 (9H, s, C(C*H*_3_)_3_), 0.03 (6H, s, Si(C*H*_3_)_2_).

### 3.18. Synthesis of trans-2-(4-Benzyloxybut-1-enyl)-6-(2-hydroxyethyl)tetrahydropyran-4-one *(**17**)*

Potassium (*E*)-(4-(-benzyloxy)but-1-en-1-yl)trifluoroborate (0.105 g, 0.39 mmol) was reated with 2-[2-(*tert*-Butyldimethylsilanyloxy)ethyl]-2,3-dihydropyran-4-one (**8**) (0.050 g, 0.195 mmol) under the standard conditions. The crude residue was treated with TBAF (0.43 mL, 1M in THF) in THF (2 mL). After stirring for 1 h, a saturated solution of NH_4_Cl was added and the mixture extracted with Et_2_O (3 × 10 mL). Combined organic extracts were dried (MgSO_4_) and concentrated *in vacuo.* The residue was purified by flash column chromatography on silica gel (eluting with CH_2_Cl_2_:methanol 9:1) to afford the title compound as a yellow oil (0.048 g, 81% yield). 

R_f_ (CH_2_Cl_2_:methanol 9:1); 0.46; ν_max_ (neat)/cm^−1^; 3342 (O-H), 2930, 2858 (C-H), 1472, 1463 (C=C), 1254, 1094 (C-O); δ_H_ (300 MHz; CDCl_3_); 7.37–7.27 (5H, m, Ar*H*), 5.71 (1H, dt, *J* = 15.8, 6.4 Hz, CH_2_C*H*CH), 5.58 (1H, dd, *J* = 15.8, 4.7 Hz, CH_2_CHC*H*), 4.75 (1H, dd, *J* = 9.7, 4.7 Hz, CHC*H*O), 4.49 (2H, s, ArCH_2_O), 4.26 (1H, m, OC*H*CH_2_), 3.73 (1H, br.s, O*H*), 3.50 (2H, t, *J =* 6.6 Hz, OC*H*_2_CH_2_), 2.67 (1H, dd, *J* = 14.5, 6.2 Hz, C*H*HCOCHH), 2.52 (1H, ddd, *J =* 14.6, 3.8, 1.4 Hz, CHHCOCH*H*), 2.43–2.28 (4H, m, CH*H*COC*H*H, C*H*_2_CHCH), 1.91–1.62 (2H, m, OCHC*H*_2_); δ_C_ (75.5 MHz; CDCl_3_); 206.5, 138.3, 132.2, 130.3, 128.4, 127.7, 127.6, 73.0, 72.9, 70.5, 69.2, 60.3, 47.6, 44.9, 37.5, 32.9.

### 3.19. Synthesis of (2S,6S)-2-Dec-1-enyl-6-phenyltetrahydropyran-4-one *(**18**)*

Potassium decenyl trifluoroborate salt (0.057 g, 0.23 mmol) was reacted with (*S*)-2-phenyl-2,3-dihydropyran-4-one ((*S*)-**3**) (0.020 g, 0.115 mmol) under the standard conditions. The crude residue was purified by flash column chromatography on silica gel (eluting with petrol:ethylacetate 9:1) to afford the title compound as a yellow oil (0.027 g, 72% yield).

R_f_ (petrol:ethyl acetate, 4:1); 0.69; ν_max_ (CH_2_Cl_2_)/cm^−1^; 3037, 2924, 2854 (C-H), 1720 (C=O), 1667 (C=C), 1603 (C=C aryl), 1249, 1052 (C-O); δ_H_ (300 MHz; CDCl_3_); 7.38–7.29 (5H, m Ar*H*), 7.00 (1H, dt, *J* = 15.7, 6.3 Hz, CH_2_C*H*), 5.57 (1H, dd, *J* = 15.7, 4.9 Hz, CH_2_CHC*H*), 5.11 (1H, dd, *J* = 7.4 Hz, 5.4 Hz, ArC*H*O, 5.71 (1H, dd, *J =* 9.6, 4.7 Hz, CHC*H*O), 2.74 (1H, dd, *J* = 14.4, 5.9 Hz, C*H*HCOCHH), 2.70 (2H, d, *J =* 6.6 Hz, CH*H*COC*H*H), 2.60 (1H, dd, *J* = 14.4, 4.6 Hz, CHHCOCH*H*), 2.06 (2H, q, *J =* 6.9 Hz, C*H*_2_CH), 1.35 (2H, dd, *J =* 13.0, 5.9 Hz, C*H*_2_CH_2_CH), 1.30–1.22 (10H, m, CH_3_(C*H*_2_)_8_), 0.87 (3H, t, *J* = 6.6 Hz, C*H*_3_); δ_C_ (75.5 MHz; CDCl_3_); 206.9, 140.0, 136.0, 128.8, 128.3, 128.1, 126.5, 73.1, 72.9, 48.0, 45.4, 32.5, 31.9, 29.5, 29.4, 29.2, 29.0, 22.8, 14.2; HPLC (Chiralcel ODH: 98:2 Hexanes:propan-2-ol, 1.0 mL·min^−1^, t_R_ = 19.43 min (minor) and 21.42 min (major).

### 3.20. Synthesis of (2S,6S)-2-(4-Bromophenyl)-6-phenyltetrahydropyran-4-one *(**19**)*

4-Bromophenylboronic acid (0.058 g, 0.287 mmol) was reacted with (*S*)-2-phenyl-2,3-dihydropyran-4-one ((*S*)-**3**) (0.025 g, 0.144 mmol) under the standard conditions. The crude residue was purified by flash column chromatography on silica gel (eluting with petrol:ethylacetate 9:1) to afford the title compound as a colourless oil (0.044 g, 93% yield).

R_f_ (petrol:ethyl acetate, 4:1); 0.61; ν_max_ (neat)/cm^−1^; 2983, 2896 (C-H), 1719 (C=O), 1596, 1494 (C=C aryl), 1245, 1231 (C-O); δ_H_ (300 MHz; CDCl_3_); 7.41 (2H, d, *J* = 7.9 Hz, Ar*H*), 7.30–7.24 (5H, m, Ar*H*), 7.17 (2H, d, *J =* 8.3 Hz, Ar*H*), 5.04 (1H, t, *J =* 5.8 Hz, ArC*H*O), 4.98 (1H, t, *J* = 5.9 Hz, ArC*H*O), 2.85 (1H, dd, *J* = 14.8, 6.5 Hz, C*H*HCOCHH), 2.76 (1H, dd, *J =* 14.8, 5.8 Hz, CHHCOCH*H*), 2.75 (2H, *J* = 6.8 Hz, CH*H*COC*H*H); δ_C_ (75.5 MHz; CDCl_3_); 206.3, 139.6, 139.0, 131.9, 128.8, 128.5, 128.3, 126.8, 122.2, 73.8, 72.9, 46.9, 46.3; HRMS (ESI^+^) calcd for C_17_H_15_BrNaO_2_ [M+Na]^+^ *m/z* 353.0153 found: *m/z* 252.0124; HPLC (Chiralcel OJ, 9:1 Hexanes:propan-2-ol, 1.0 mL·min^−1^, t_R_ = 20.36 min (minor) and 28.61 min (major).

## 4. Conclusions 

In summary, the catalytic conjugate addition of both aryl- and alkenylboronates to dihydropyranone templates have been accomplished in high yields, leading to the selective synthesis of 2,6-*trans*-tetrahydropyran derivatives. The selective formation of the 2,6-*trans*-tetrahydropyran stereoisomer is consistent with a mechanism involving alkene association and carbometalation on the less hindered face of the dihydropyranone. This methodology has simultaneously expanded the limited precedent for metal-catalysed addition of organoboron reagents to enantioenriched substrates and demonstrated the utility of sequential catalysis in the construction of “natural product-like” molecules.
